# Genomic analysis of six new *Geobacillus* strains reveals highly conserved carbohydrate degradation architectures and strategies

**DOI:** 10.3389/fmicb.2015.00430

**Published:** 2015-05-12

**Authors:** Phillip J. Brumm, Pieter De Maayer, David A. Mead, Don A. Cowan

**Affiliations:** ^1^C5•6 TechnologiesMiddleton, WI, USA; ^2^Great Lakes Bioenergy Research Center, University of WisconsinMadison, WI, USA; ^3^Centre for Microbial Ecology and Genomics, Genomics Research Institute, University of PretoriaPretoria, South Africa; ^4^Department of Microbiology and Plant Pathology, University of PretoriaPretoria, South Africa; ^5^Lucigen CorporationMiddleton, WI, USA

**Keywords:** xylan, *Geobacillus*, galactose, arabinan, starch, genome sequencing, biomass, metabolism

## Abstract

In this work we report the whole genome sequences of six new *Geobacillus* xylanolytic strains along with the genomic analysis of their capability to degrade carbohydrates. The six sequenced *Geobacillus* strains described here have a range of GC contents from 43.9% to 52.5% and clade with named *Geobacillus* species throughout the entire genus. We have identified a ~200 kb unique super-cluster in all six strains, containing five to eight distinct carbohydrate degradation clusters in a single genomic region, a feature not seen in other genera. The *Geobacillus* strains rely on a small number of secreted enzymes located within distinct clusters for carbohydrate utilization, in contrast to most biomass-degrading organisms which contain numerous secreted enzymes located randomly throughout the genomes. All six strains are able to utilize fructose, arabinose, xylose, mannitol, gluconate, xylan, and α-1,6-glucosides. The gene clusters for utilization of these seven substrates have identical organization and the individual proteins have a high percent identity to their homologs. The strains show significant differences in their ability to utilize inositol, sucrose, lactose, α-mannosides, α-1,4-glucosides and arabinan.

## Introduction

Thermophiles have been a source of industrial enzymes for over 30 years (Vieille and Zeikus, 2001; Haki and Rakshit, 2003; de Miguel Bouzas et al., 2006). A range of industrial applications including paper manufacturing, brewing, biomass deconstruction and the production of animal feeds (Dersjant-Li et al., 2001; Tricarico and Dawson, 2005; Valls and Roncero, 2009; Valls et al., 2010) have used thermophilic enzymes for the degradation of xylan. Xylans are the most abundant form of hemicellulose (Saha, 2003). The defining feature of xylans is a backbone of beta-1,4-linked xylose residues. While cellulose is a homopolymer of beta-1,4-linked glucose, xylans are heteropolymers containing a range of species-specific modifications to the backbone chain (Saha, 2003). These modifications include the attachment of neutral sugars such as arabinose, galactose, and glucose, attachment of charged sugars such as glucuronic acid, and acetylation, giving rise to unsubstituted xylans, arabinoxylans, glucuronoxylans, and arabinoglucuronoxylans (these will all be collectively called xylan). The result of these modifications is a bewildering diversity in the chemical compositions and structures of xylans (recently reviewed in Girio et al., 2010), and the need for a wide range of enzymes and enzyme activities to degrade these structures. As a result, many enzymes active on xylan have been isolated and characterized from a wide range of organisms, especially thermophilic bacteria. *Geobacillus stearothermophilus* (previously known as *Bacillus stearothermophilus*, Nazina et al., 2001) is a heavily studied source of many xylan-degrading enzymes. Xylan-degrading enzymes characterized from *Geobacillus stearothermophilus* strain T-6 include two xylanases (Teplitsky et al., 2004; Solomon et al., 2007), an α-glucuronidase (Choi et al., 2000), three xylosidases (Bravman et al., 2003; Czjzek et al., 2004; Brux et al., 2006), one arabinofuranosidase, and one arabinopyranosidase (Shallom et al., 2002; Salama et al., 2012). Other *Geobacillus* species have been identified as sources of thermostable xylanase (Gerasimova and Kuisiene, 2012; Liu et al., 2012; Verma and Satyanarayana, 2012; Anand et al., 2013; Bhalla et al., 2014), with all the enzymes showing properties similar to those of the *G. stearothermophilus* enzyme. A range of other enzymes with potential industrial applications have been identified in *Geobacillus* species including α-galactosidases (Fridjonsson et al., 1999; Merceron et al., 2012) for use in soy processing, β-galactosidases (Goodman and Pederson, 1976; Hirata et al., 1984, 1986; Solomon et al., 2013) for use in milk processing, lipases (Jeong et al., 2001; Sinchaikul et al., 2002; Abdul Rahman et al., 2009; Ebrahimpour et al., 2011; Balan et al., 2012) and proteases (Nishiya and Imanaka, 1990; Jang et al., 1992; Hawumba et al., 2002; Chen et al., 2004; Itoi et al., 2006) for use in detergents, and amylases (Sen and Oriel, 1989; Brumm et al., 1991; Narang and Satyanarayana, 2001; Kamasaka et al., 2002; Ferner-Ortner-Bleckmann et al., 2009; Mok et al., 2013; Nasrollahi et al., 2013) for use in corn wet milling, baking and ethanol production.

A 23.55 kb genomic DNA fragment from *Geobacillus stearothermophilus* strain T-6 contains the genes for extracellular and intracellular xylanases, β-xylosidase, and 12 genes involved in transport and metabolism of glucuronic acid (Shulami et al., 1999). The organization of the arabinan utilization genes from this organism, which form a separate cluster contiguous to the xylan utilization cluster, was described later (Shulami et al., 2011). A complete genome sequence for *G. stearothermophilus* strain T-6 has not been published, resulting in only limited understanding of the organization of xylan and arabinan metabolism within the *G. stearothermophilus* genome. Without a complete genome, it is also unclear if the genes present in these two clusters represent the complete set of genes needed for pentosan degradation. Without complete genome sequences, it is impossible to determine the genomic context of the individual enzymes described above, and if these individual enzymes are present at the genus, species, or strain level.

Whole genome sequencing is a potent tool for understanding the collection of genes a microorganism utilizes for carbohydrate degradation (Suen et al., 2011; Mead et al., 2012, 2013; Christopherson et al., 2013). To date, only a limited number of complete *Geobacillus* genomes have been published including *G. thermodenitrificans* (Feng et al., 2007; Yao et al., 2013), *G. kaustophilus* (Takami et al., 2004), *Geobacillus* sp. strain GHH01 (Wiegand et al., 2013), *Geobacillus* sp. strain JF8 (Shintani et al., 2014), *G. thermoglucosidans* TNO-09.020 (Zhao et al., 2012), and *G. thermoleovorans* CCB_US3_UF5 (Muhd Sakaff et al., 2012), and no detailed analysis of the carbohydrate degradation systems of these organisms have been published. Our group has isolated six novel xylanolytic *Geobacillus* strains as part of an effort to identify new, high specific activity thermophilic enzymes. The genomes of all six strains have been determined, with five of the six genome sequences deposited in GenBank, and the sixth available via the JGI genome portal. Using these genome resources, the carbohydrate degradation clusters in these six strains were identified and compared. The results of this analysis revealed that both the organization and the individual genes of carbohydrate metabolism are highly conserved throughout the genus. In addition, many of these carbohydrate degradation clusters reside in a single, 200-kb conserved genome region.

## Materials and methods

The azurine cross-linked-labeled (AZCL) polysaccharide AZCL-Arabinoxylan (AZCL-AX) and was obtained from Megazyme International (Wicklow, Ireland). 4-Methylumbelliferyl-β-D-cellobioside (MUC) and 4-methylumbelliferyl-β-D-xylopyranoside (MUX), were obtained from Research Products International Corp. (Mt. Prospect, IL, USA). CelLytic IIB reagent, birchwood xylan, arabinogalactan from larch wood (Fluka) and 4-methylumbelliferyl-α-D-arabinofuranoside (MUA) were purchased from Sigma-Aldrich (St. Louis, MO, USA). Xylo-oligosaccharides were obtained from Cascade Analytical Reagents and Biochemicals (Corvallis, OR, USA). All other chemicals were of analytical grade.

*Geobacillus* strains were isolated from environmental samples (Table 1) on YTP-2 agar (contains (per liter) 2.0 g yeast extract, 2.0 g tryptone, 2.0 g sodium pyruvate, 1.0 g KCl, 2.0 g KNO3, 2.0 g Na_2_HPO_4_.7H_2_O, 0.1 g MgSO_4_, 0.03 g CaCl_2_, 8.0 g agar, and 2.0 ml clarified tomato juice) at 70°C as described previously (Mead et al., 2012). For preparation of genomic DNA, 1 liter cultures of *Geobacillus* isolates were grown from a single colony in YTP-2 medium at 70°C in flasks agitated at 200 rpm for 18 h and collected by centrifugation. The cell concentrate was lysed using a combination of SDS and proteinase K, and genomic DNA was isolated using a phenol/chloroform extraction (Sambrook et al., 1989). The genomic DNA was precipitated, and treated with RNase to remove residual contaminating RNA.

**Table 1:** ***Geobacillus* genomes sequenced in this work**.

	**MC52, MC61, YS93**	**1MC16**	**56T2**	**56T3**
Source	Obsidian hot spring WY, USA	Grass compost WI, USA	Double hot springs NV, USA	Sandy's spring west NV, USA
Latitude	44.376262	43.111566	41.051289	40.651893
Longitude	−110.690383	−89.518892	−119.028790	−119.376659
Temperature	79°C	60°C	79.6°C	80°C
pH	6.7	unknown	8.0	7.4

Cultures for enzyme assays were grown in 1.0 ml of YT2 medium (contains (per liter) 2.0 g yeast extract, 2.0 g tryptone, 2.0 g carbohydrate substrate, 1.0 g KCl, 2.0 g KNO_3_, 2.0 g Na_2_HPO_4_.7H_2_O, 0.1 g MgSO_4_, 0.03 g CaCl_2_, 8.0 g agar, and 2.0 ml clarified tomato juice). Cultures were grown from single colonies at 70°C in 2.0 ml screw-cap vials for 72 h at 1000 rpm in a Thermomixer R (Eppendorf, Hamburg, Germany). Cells were recovered by centrifugation, and the cell pellets were lysed by treatment with 0.1 ml of CelLytic IIB reagent. Qualitative *endo*-activities of supernatant and lysate samples were determined in 0.50 ml of 50 mM acetate buffer, pH 5.8, containing 0.2% AZCL insoluble substrates and 50 μl of supernatant or 10 μl of clarified lysate. Assays were performed overnight at 70°C, with shaking at 1000 rpm in a Thermomixer R. Tubes were clarified by centrifugation, and absorbance values at 600 nm were determined using a Bio-Tek ELx800 plate reader. The *exo*-activities of supernatant and lysate samples were determined by spotting 5.0 μl of clarified lysate directly on agar plates containing 10 mM 4-methylumbelliferyl substrate. Plates were incubated in a 70°C incubator for 2 h; after incubation, the plates were examined using a hand-held UV lamp and compared with negative and positive controls. Duplicate cultures were used for all assay experiments.

The genomes of six *Geobacillus* isolates were sequenced at the Joint Genome Institute (JGI) using Sanger sequencing with a combination of 6 kb and 34 kb DNA libraries and 454 FLX pyrosequencing done to a depth of 20× coverage; Solexa sequencing data was used to polish the assemblies. All general aspects of library construction and sequencing performed at the JGI can be found at their website. The Phred/Phrap/Consed software package (Lee and Vega, 2004; Machado et al., 2011) was used to assemble 6-kb and fosmid libraries. Genes were identified using Prodigal (Hyatt et al., 2010) as part of the Oak Ridge National Laboratory genome annotation pipeline, followed by a round of manual curation using the JGI GenePRIMP pipeline (Pati et al., 2010). The predicted CDSs were translated and used to search the National Center for Biotechnology Information (NCBI) non-redundant protein database, UniProt, TIGRFam, Pfam, PRIAM, KEGG, COG, and InterPro databases. These data sources were combined to assert a product description for each predicted protein. Non-coding genes and miscellaneous features were predicted using tRNAscan-SE (Lowe and Eddy, 1997), RNAMMer (Lagesen et al., 2007), Rfam (Griffiths-Jones et al., 2003), TMHMM (Chen et al., 2003), and signalP (Krogh et al., 2001). The *Geobacillus* cultures are available from the Bacillus Genetics Stock Center (BGSC) at Ohio State University; all genome sequences can be accessed online (Table 1).

The phylogeny of the novel *Geobacillus* strains was determined using the 16S rRNA gene sequences of the six sequenced strains, as well as those of the type strains of all validly described *Geobacillus* spp. The 16S rRNA gene sequences were aligned using MUSCLE (Edgar, 2004), pairwise distances were estimated using the Maximum Composite Likelihood (MCL) approach, and initial trees for heuristic search were obtained automatically by applying the Neighbour-Joining method in MEGA 5 (Tamura et al., 2011). The alignment and heuristic trees were then used to infer the phylogeny using the Maximum Likelihood method based on the Tamura-Nei (Tamura and Nei, 1993).

Carbohydrate utilization enzymes were identified from UniProt (Apweiler et al., 2004; Consortium, 2013, 2014), and BLASTp analysis (Cameron et al., 2004) was used to identify orthologs in the genomes. Neighborhood analysis was performed using IMG tools (Markowitz et al., 2012) to determine clusters and manually curate the electronic annotations.

## Results

As part of a project to identify new thermophilic enzymes that degrade biomass, microbial cultures from hot springs and composts were isolated and biochemically screened to identify novel, aerobic, biomass-degrading thermophiles. Aerobic enrichments were performed at 70°C, and the vast majority of the 100 isolates were *Geobacillus* or *Thermus* species. Six of these *Geobacillus* isolates were selected for additional characterization based on the ability of colonies to hydrolyze MUX or MUC incorporated into agar plates. Five of these isolates were from hot springs in the United States (Yellowstone National Park and Nevada) and one was from a grass compost sample collected in Middleton WI (Table 1).

To determine if these isolates produced xylan degrading enzymes, the six selected cultures (designated C56-YS93 (YS93), G11MC16 (1MC16), Y412MC52 (MC52), Y412MC61 (MC61), C56-56T2 (56T2), and C56-T3 (56T3) were grown in 1.0 ml cultures of YT2 media containing one of six carbohydrate substrates (pyruvate, glucose, xylose, arabinose, xylo-oligosaccharides and arabinogalactan) and assayed qualitatively for the production and activity of extracellular xylanase and intracellular β-xylosidase as described in Materials and Methods. All six strains produced extracellular xylanase when grown on either xylose or pyruvate (Table 2). In addition, extracellular xylanase was produced by at least three of the cultures when grown on arabinose, arabinogalactan or xylo-oligosaccharides. None of the six strains produced extracellular xylanase when grown on glucose, in agreement with reports of catabolite repression of *G. stearothermophilus* extracellular xylanase production (Cho and Choi, 1999). Intracellular β-xylosidase was produced by all six strains when grown on xylose while none of the strains produced intracellular β-xylosidase when grown on pyruvate or glucose. Only one strain (YS93) produced intracellular β-xylosidase when grown on arabinose, arabinogalactan and xylo-oligosaccharides. The results of the extracellular and intracellular assays confirmed that all six strains possess the ability to degrade xylan.

**Table 2:** **Enzymatic activities of *Geobacillus* strains grown on various carbohydrate substrates**.

**Strain**	**Pyruvate**	**Xylose**	**Glucose**	**Arabinose**	**XO^a^**	**AG^b^**
**EXTRACELLULAR ENZYMATIC ACTIVITY**						
YS93	xylanase	xylanase	n.d.	xylanase	xylanase	xylanase
1MC16	xylanase	xylanase	n.d.	xylanase	xylanase	n.d.
MC52	xylanase	xylanase	n.d.	n.d.	xylanase	xylanase arabinase
MC61	xylanase	xylanase	n.d.	n.d.	xylanase	xylanase arabinase
56T2	xylanase	xylanase	n.d.	xylanase	n.d.	xylanase
56T3	xylanase	xylanase	n.d.	n.d.	n.d.	n.d.
**INTRACELLULAR ENZYMATIC ACTIVITY**						
YS93	n.d.	xylosidase	n.d.	xylosidase	xylosidase	xylosidase
1MC16	n.d.	xylosidase	n.d.	n.d.	n.d.	n.d.
MC61	arabinosidase	xylosidase	n.d.	n.d.	xylosidase	arabinosidase
MC52	arabinosidase	xylosidase	n.d.	n.d.	xylosidase	arabinosidase
56T2	n.d.	xylosidase	n.d.	n.d.	n.d.	n.d.
56T3	n.d.	xylosidase	n.d.	n.d.	n.d.	n.d.
YS93	n.d.	xylosidase	n.d.	xylosidase	xylosidase	xylosidase

*Geobacillus strains grown and assayed as described in Methods*.

a *XO, xylo-oligosaccharides*.

b *AG, arabinogalactan*.

*n.d. – none detected*.

Based on the positive results obtained in the enzyme screening experiments, the six strains were submitted for sequencing by the JGI of the Department of Energy. Genome sequencing yielded five closed genomes with one isolate, 1MC16, left as a permanent draft genome containing 31 contigs (Table 3). The genomes are all of similar size, ranging from 3.5 to 4.0 megabases. Plasmid content varies from none in 56T3, one in MC52 and MC61, and two in strains YS93 and 56T2. The presence of plasmids in 1MC16 could not be confirmed from the assembled contigs. The genomes display significantly different G+C contents. YS93 has a mean genomic G+C content of 43.9%, 1MC16 has an intermediate value of 48.8% G+C, and MC52, MC61, 56T2, and 56T3 have significantly higher values of 52.3–52.5% G+C (Table 3).

**Table 3:** ***Geobacillus* sequencing results**.

***Geobacillus* species**	**Genome size**	**Genome contigs**	**Plasmids**	**GC content**	**GenBank ID**
YS93	3,993,793	1	2	43.9	NC_015660
1MC16	3,545,187	31	n.d.	48.8	ABVH01000001-ABVH01000031
MC52	3,673,940	1	1	52.3	NC_014915
MC61	3,667,901	1	1	52.3	NC_013411
56T2	3,545,944	1	2	52.4	NA^*^
56T3	3,650,813	1	0	52.5	NC_014206

*Geobacillus strains isolated and sequenced as described in Methods*.

* *Genome available at http://gp-next.jgi-psf.org:1090/GeospC56T2/GeospC56T2.info.html*.

**Figure 1:**
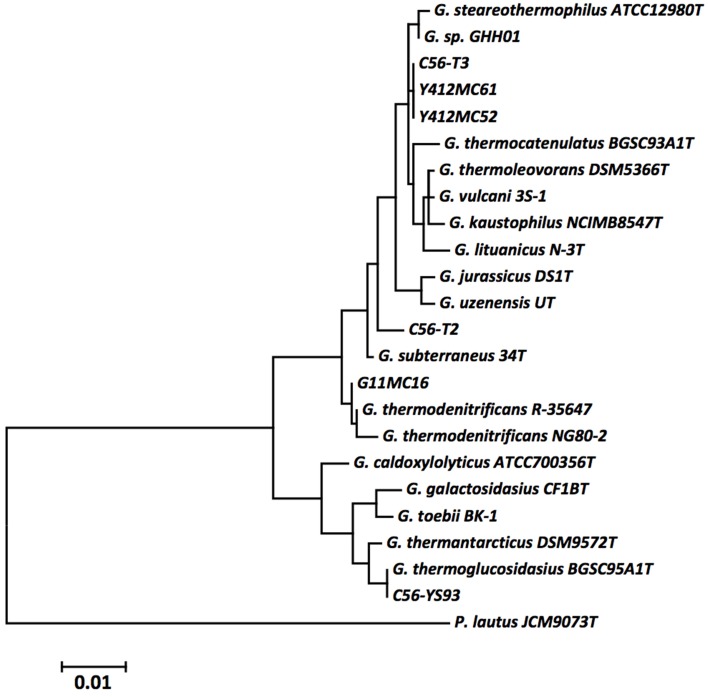
**The evolutionary history was inferred by using the Maximum Likelihood method based on the Tamura-Nei model (Tamura and Nei, 1993)**. The tree with the highest log likelihood (−3118.4467) is shown. Initial tree(s) for the heuristic search were obtained automatically by applying Neighbor-Join and BioNJ algorithms to a matrix of pairwise distances estimated using the Maximum Composite Likelihood (MCL) approach, and then selecting the topology with superior log likelihood value. The tree is drawn to scale, with branch lengths measured in the number of substitutions per site. The analysis involved 24 nucleotide sequences. All positions containing gaps and missing data were eliminated. There were a total of 1260 positions in the final dataset. Evolutionary analyses were conducted in MEGA5 (Tamura et al., 2011). The type strains of all validly described species are included (NCBI accession numbers): *G. caldoxylolyticus* ATCC700356^T^ (AF067651), *G. galactosidasius* CF1B^T^ (AM408559), *G. jurassicus* DS1^T^ (FN428697), *G. kaustophilus* NCIMB8547^T^ (X60618), *G. lituanicus* N-3^T^ (AY044055), *G. stearothermophilus* R-35646^T^ (FN428694), *G. subterraneus* 34^T^ (AF276306), *G. thermantarcticus* DSM9572^T^ (FR749957), *G. thermocatenulatus* BGSC93A1^T^ (AY608935), *G. thermodenitrificans* R-35647^T^ (FN538993), *G. thermoglucosidasius* BGSC95A1^T^ (FN428685), *G. thermoleovorans* DSM5366^T^ (Z26923), *G. toebii* BK-1^T^ (FN428690), *G. uzenensis* U^T^ (AF276304), and *G. vulcani* 3S-1^T^ (AJ293805). The 16S rRNA sequence of *Paenibacillus lautus* JCM9073^T^ (AB073188) was used to root the tree.

A phylogenetic tree of 16S rRNA gene sequences was constructed using the Maximum Likelihood method based on the Tamura-Nei model (Tamura and Nei, 1993) to determine the phylogenetic positions of the novel strains. The resulting tree (Figure 1) shows YS93 clades with *G. thermoglucosidasius*, 1MC16 clade with *G. thermodenitrificans*, MC52, MC61, and 56T3 clade with *G. stearothermophilus* and *G. thermocatenulatus*, and 56T2 may represent a novel species of *Geobacillus*. To confirm the assignments obtained with 16S rRNA gene sequences, pairwise average nucleotide identity values were calculated for the six strains against all draft, permanent draft, and finished Geobacillus genomes in the IMG database. Average nucleotide identity values (ANI) (Kim et al., 2014) were calculated using software developed for the IMG (Markowitz et al., 2006, 2014). The results (Table 4) confirm the classification of the strains obtained using 16S rRNA gene sequences. YS93 clades with other G. thermoglucosidasius strains (pink), 1MC16 clades with *G. thermodenitrificans* strains (blue), 12MC52, 12MC61, and 56T3 clade together in what appears to be a new species (yellow), and 56T2 appears to clade only with itself (gray).

**Table 4:**
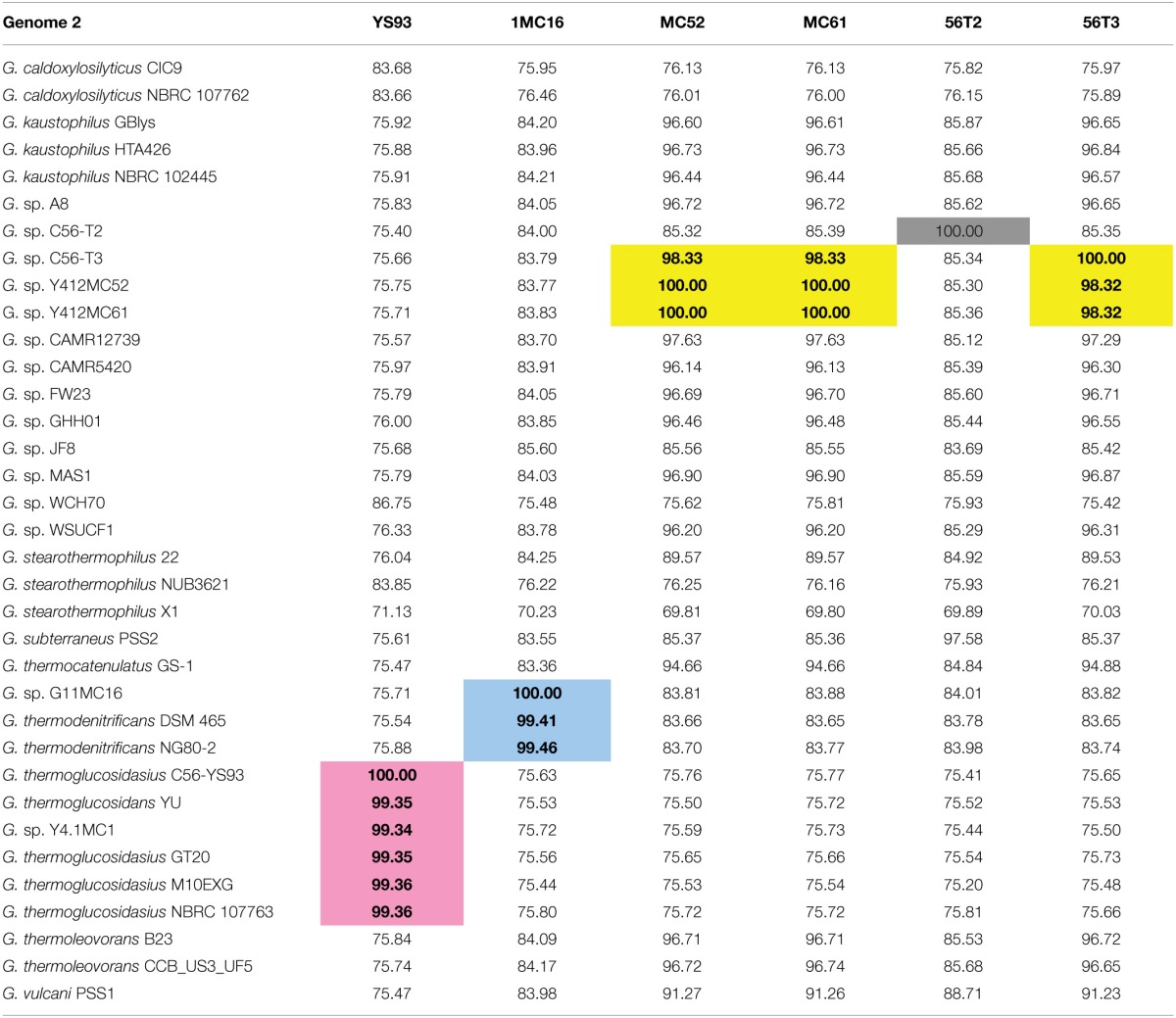
***Geobacillus* average nucleotide identity (ANI) results**.

*Strains forming a clade with YS93 are shown in bold and highlighted in pink, strains forming a clade with 1MC16 are shown in bold and highlighted in blue, the clade formed by 12MC52, 12MC61, and 56T3 is shown in bold and highlighted in yellow, and 56T2 is highlighted in gray*.

## Identification of metabolic clusters

**Figure 2:**
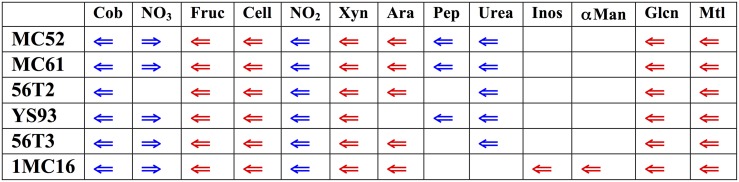
**Diagram of major functional clusters found in the conserved regions; carbohydrate utilization clusters are shown in red, non-carbohydrate clusters in blue**. Cob, cobalamin biosynthetic cluster, NO_3_, nitrate reductase cluster; Fruc, fructose utilization cluster; Cell, cellobiose utilization cluster; NO2_3_, nitrite reductase cluster; Xyn, xylose and xylan utilization cluster; Ara, arabinose and arabinan utilization cluster, and ribose transporter cluster: Pep, peptide utilization cluster; Urea, urease and urea utilization cluster, Inos, inositol-phosphate utilization cluster; αMan, α-mannoside utilization cluster; GLcn gluconate utilization cluster; Mtl, mannitol utilization cluster. The gene sequence values for the corresponding genomes regions are (start-end): MC61, 2635441-2855821; MC52, 1775380-1995757; 56T2, 1737107-1912324; 56T3, 1646809-1858633; YS93, 2080803-2255158; 1MC16, contig ABVH01000004 28446-229812.

The six genomes were searched for the location of orthologs of the xylan cluster described in *G. stearothermophilus* T-6. Surprisingly, in all six strains the xylan utilization cluster is located in a similar, highly conserved region of the *Geobacillus* genomes (Figure 2). In all six strains, this genome region contains clusters for the utilization of xylan as well as fructose, cellobiose, gluconate, and mannitol utilization clusters. In five of the six strains, clusters for the utilization of arabinan, arabinose, and ribose are also present in this region. Inositol and α-mannoside utilization clusters are present this region in one strain. In addition to carbohydrate utilization clusters, all six strains possess a 16-gene biosynthesis cobalamin cluster and a 4-gene nitrite reductase cluster. Five of the six strains contain a 13-gene urea utilization cluster and a 4-gene nitrate reductase cluster. This large super-cluster of metabolic clusters, conserved at the genus level, appears to be a unique feature of the *Geobacillus*. Carbohydrate utilization clusters found in this ~200 kb region of the genomes will be described first, proceeding in the direction of transcription. Following these descriptions, carbohydrate utilization clusters not found in the ~200 kb region will be described.

## Carbohydrate clusters found in the ~200 Kb region of the sequenced geobacillus strains

### Mannitol metabolism

In all six strains, orthologous clusters code for three-component phosphotransferase system (PTS) that uses phosphoenolpyruvate to transport the sugar into the cell and phosphorylate it, generating intracellular mannitol-1-phosphate. A MtlR family transcriptional regulator controls mannitol uptake in all six strains. The six mannitol utilization clusters also contain a gene coding for mannitol-1-phosphate 5-dehydrogenase, which converts the mannitol-1-phosphate to fructose-1-phosphate. Similar transport and metabolism clusters are used for fructose, cellobiose and sucrose metabolism.

### Gluconate metabolism

All six strains possess an orthologous cluster for gluconate utilization similar to the GntU, GntK, and GntR cluster found in *E. coli* (Tong et al., 1996). Unlike the *B. subtilis* gluconate utilization cluster (Reizer et al., 1991), the *Geobacillus* cluster does not include a GntZ gene coding for 6-phosphogluconate dehydrogenase. The GntZ gene is present in all six strains, located randomly throughout the genomes.

### α-mannosides and inositol-phosphate utilization

Only one of the six strains, 1MC16, possesses the ability to utilize either inositol-phosphates or α-mannosides. The two clusters are located upstream of the gluconate cluster, where the other five *Geobacillus* genomes contain a 13-gene urease/urea utilization cluster. The mannoside utilization cluster has a 3-component ABC transporter system and an intracellular α-mannosidase, all under the control of a GntR family transcriptional regulator. Orthologous mannosidase clusters are present in the genomes of *G. thermodenitrificans* DSM 465 and *G. thermodenitrificans* NG80-2 (Feng et al., 2007), and the individual genes of these two strains are 99–100% identical to their 1MC16 gene counterparts.

**Figure 3:**
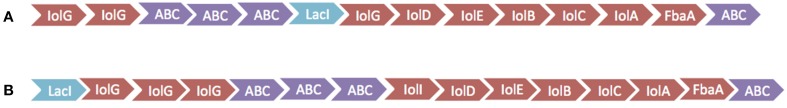
**Diagram of inositol utilization clusters. (A)** Cluster found in 1MC16, *G. thermodenitrificans* DSM 465 and *G. thermodenitrificans* NG80-2. **(B)** Cluster found in *G. kaustophilus* HTA426, *Geobacillus subterraneus* PSS2, *G. thermoglucosidasius* M10EXG, and *G. thermocatenulatus* GS-1.

The inositol-phosphate utilization cluster (Table 5) has two separate parts. The first is a five-gene cluster containing a 3-component ABC transporter system, inositol 2-dehydrogenase, and an oxidoreductase domain protein. Following this is an inositol metabolic gene cluster of iolG, iolD, iolE, iolB, iolC, and iolA, all under the control of a LacI family transcriptional regulator. Identical inositol-phosphate utilization clusters are present in *G. thermodenitrificans* DSM 465 and *G. thermodenitrificans* NG80-2. Other *Geobacillus* species possess similar clusters, but are organized with the three-genes of the ABC transporter following the genes for iolG (Yoshida et al., 2012) and with an additional protein, IolI, 2-keto-*myo*-inositol isomerase (Figure 3).

**Table 5:** **1MC16 Inositol-phosphate metabolic cluster**.

**Annotation**	**Gene**
Inositol 2-dehydrogenase, IolG	1528
Oxidoreductase domain protein, IolG	1530
ABC transporter-related protein	1531
ABC-type transport systems, permease	1532
ABC-type sugar transport system, periplasmic component	1533
Transcriptional regulator, LacI family	1534
*myo*-Inositol 2-dehydrogenase, IolG	1535
Trihydroxycyclohexane-1,2-dione hydrolase, IolD	1536
Inosose dehydratase, IolE	1537
5-Deoxy-glucuronate isomerase, IolB	1538
5-Dehydro-2-deoxygluconokinase, IolC	1539
methylmalonate-semialdehyde dehydrogenase, IolA	1540
Fructose 1,6-bisphosphate aldolase, IolJ	1541

### Arabinose and arabinan metabolism

Unlike xylan utilization, arabinose and arabinan utilization capability is strongly strain dependent (Table 6). None of the strains possesses the complete arabinan cluster present in *G. stearothermophilus* T-6 (Shulami et al., 2011). Strain YS93 possesses none of the enzymes required for uptake and metabolism of either arabinose or arabinan. Strain 56T2 possesses the genes for metabolism of arabinose (genes 7–10 and 22–27) but none of the dedicated transporter systems. This suggests that the organism can utilize arabinose in arabinoxylan oligosaccharides that was transported into the cell by xylan transporters, but not extracellular arabinose or arabinan. The arabinose utilization cluster of strains MC52 and MC61 are most similar to the reported T-6 arabinan cluster, lacking only one of the two transporter clusters found in T-6 (genes 1–6). This suggests that these two organisms can utilize the full range of arabinose, small arabinan oligosaccharides, and linear arabinan. 1MC16 possesses two ABC transporter systems (genes 4–6 and 19–21). The first transporter system is orthologous to the *G. stearothermophilus* T-6 araT, AraE, araG transporter, while the second has no orthologs in T-6. While the three component araT, AraE, araG transporter system is annotated as an arabinose transport system, the three genes show remarkable homology to the rbsA, rbsB, rbsC cluster responsible for transport of ribose in *B. subtilis* (Woodson and Devine, 1994; Strauch, 1995), and may actually function as a ribose transport system within the arabinan-arabinose cluster.

**Table 6:** **Arabinose and ribose metabolic cluster**.

	**Annotation**	**YS93**	**1MC16**	**MC52**	**MC61**	**56T2**	**56T3**
1	Sugar ABC transporter sugar-binding protein	-	1543	-	-	-	1614
2	Multi-sensor signal transduction histidine kinase	-	1544	-	-	-	1615
3	AraC family transcriptional regulator	-	1546	-	-	-	1616
4	ABC transporter substrate-binding protein	-	1547	-	-	-	1617
5	ABC transporter	-	1548	-	-	-	1618
6	Inner-membrane translocator	-	1549	-	-	-	1619
7	GntR family transcriptional regulator	-	1550	1867	2737	1890	1620
8	L-ribulose-5-phosphate 4-epimerase	-	1551	1866	2736	1889	1621
9	L-ribulokinase	-	1552	1865	2735	1888	1622
10	L-arabinose isomerase	-	1553	1864	2734	1887	1623
11	Arabinopyranosidase	-	-	1863	2733	-	-
12	Intracellular endo-α- (1-5)-L-arabinanase	-	-	1862	2732	-	-
13	Family 1 extracellular solute-binding protein	-	-	1861	2731	-	-
14	Binding-protein-dependent transporters inner membrane protein	-	-	1860	2730	-	-
15	Sugar ABC transporter permease	-	-	1859	2729	-	-
16	Extracellular arabinanase	-	-	1858	2728	-	-
17	α-L-arabinofuranosidase	-	-	1857	2727	-	-
18	Unknown 88 a.a. protein	-	-	1856	2726	-	-
19	Family 1 extracellular solute-binding protein	-	1554	-	-	-	1624
20	Binding-protein-dependent transporters inner membrane protein	-	1555	-	-	-	1625
21	Sugar ABC transporter permease	-	1556	-	-	-	1626
22	α-N-arabinofuranosidase	-	-	-	-	1885	-
23	α-L-arabinofuranosidase	-	1557	1855	2725	1884	1627
24	Oxidoreductase domain-containing protein	-	1558	1854	2724	1883	1628
25	Aldose 1-epimerase	-	1569	1853	2723	1878	1629
26	HAD-superfamily hydrolase	-	1559	1852	2722	1881	1630
27	Glycerol-1-phosphate dehydrogenase	-	1560	1851	2721	1880	1631
28	β-L-arabinofuranosidase	-	-	1850	2720	-	-

Strain 1MC16 lacks the eight-gene cluster containing the extracellular arabinanase, transporter and intracellular endo-α-arabinanase, α-L-arabinofuranosidase, and arabinopyranosidase (genes 11–18) present in the T-6 cluster. 1MC16 possesses all metabolic enzymes needed for arabinose and potentially arabinooligosaccharide metabolism (genes 7–10 and 23–27), suggesting that the organism can utilize arabinose in arabinoxylan oligosaccharides, extracellular arabinose and possibly small arabinan oligosaccharides.

The most complex arabinose metabolic system is present in 56T3. 56T3 possesses an arabinose cluster that is orthologous to the 1MC16 cluster described above. However, in addition to this cluster, 56T3 possesses a seven-gene arabinan-utilization cluster consisting of a transcription regulator, three-component ABC transporter system, and three intracellular proteins, an arabinase, an arabinofuranosidase, and an annotated oxidoreductase with unknown function (Table 7). This cluster is located adjacent to the galactose utilization cluster in 56T3 and it is not orthologous to the clusters found in T-6, MC52, and MC61, but is closely related to the cluster found in the unpublished genome of *Geobacillus* sp. MAS1 (NCBI/RefSeq: AYSF01000001 through AYSF01000006) as well as distantly related to clusters in *Bacillus* spp. and *Anoxybacillus tepidamans* PS2.

**Table 7:** **56T3 Arabinose and arabinan metabolic cluster**.

**Annotation**	**Gene**
Transcriptional regulator, ArsR family	1352
Extracellular solute-binding protein family 1	1353
Binding-protein-dependent transport systems inner membrane component	1354
Binding-protein-dependent transport systems inner membrane component	1355
GH43 Intracellular endo-α-(1-5)-L-arabinanase	1356
GH2 α-L-arabinofuranosidase	1357
Oxidoreductase	1358
Galactokinase, GalK	1361
UDP-glucose 4-epimerase, GalE	1362
Gal-1-phosphate uridylyltransferase, GalT	1363
Transcriptional regulator, LacI family	1364

### Xylose and xylan metabolism

As expected from the fermentation results, all six strains possess gene clusters for xylan degradation and metabolism. Xylose and xylan are transported and metabolized by all six strains via a large single cluster containing as many as 32 genes (De Maayer et al., 2014) (Table 8). A single secreted xylanase (XynA) degrades xylan into oligosaccharides. Two, three-gene ABC transporters of xylose and xylooligosaccharides are present in all six strains (shown in bold, genes 3, 4, 5 and 10, 11, 12). In addition, strains 56T2 and C56-T3 contain a third three-gene ABC transporter (27, 28, 29). The transported oligosaccharides are further degraded into monosaccharides within the cell by an intracellular xylanase (XynA2), xylosidases (XynB and XynB2) and an α-glucuronidase (AguA) similar to those described in *G. stearothermophilus* T-6 (Shulami et al., 1999). The enzymes for glucuronate utilization are coded for within the cluster (genes 16, 18, 19, 20), as are the enzymes for xylose utilization (genes 31 and 32).

**Table 8:** **Xylose and xylan metabolic cluster**.

	**Annotation**	**YS93**	**1MC16**	**MC52**	**MC61**	**56T2**	**56T3**
1	Integral membrane sensor signal transduction histidine kinase	2272	1564	1849	2719	1877	1634
2	AraC family transcriptional regulator	2271	1565	1848	2718	1876	1635
3	Family 1 extracellular solute-binding protein	2270	1566	1846	2716	1875	1636
4	Binding-protein-dependent transporters inner membrane component	2269	1567	1845	2715	1874	1637
5	Binding-protein-dependent transporters inner membrane component	2268	1568	1844	2714	1873	1638
6	Aldose 1-epimerase	2267	1569	1843	2713	-	1639
7	Polysaccharide deacetylase	2266	1570	1842	2712	-	1640
8	Xylan 1,4-beta-xylosidase	2265	1571	1841	2711	1872	1641
9	Endo-1,4-beta-xylanase	2264	1572	1840	2710	1871	1642
10	Family 1 extracellular solute-binding protein	2262	1574	1839	2709	1873	1643
11	Binding-protein-dependent transporters inner membrane component	2261	1575	1838	2708	1874	1644
12	Binding-protein-dependent transporters inner membrane component	2260	1577	1837	2707	1875	1645
13	**α-glucuronidase**	2259	1578	1836	2706	1870	1646
14	Xylan 1,4-beta-xylosidase	2258	1579	1835	2705	1869	1647
15	PfkB domain-containing protein	2257	1580	1834	2704	-	1648
16	2-dehydro-3-deoxyphosphogluconate aldolase	2256	1581	1833	2703	1867	1649
17	GntR family transcriptional regulator	2255	1582	1832	2702	1866	1650
18	Uronate isomerase	2254	1583	-	-	-	1651
19	Mannonate dehydratase	2253	1584	1828	2699	1864	1652
20	Short-chain dehydrogenase	2252	1585	1829	2698	1863	1653
21	Hypothetical protein	2251	1586	1827	2697	-	1654
22	Endo-1,4-beta-xylanase	2250	1587	1825	2695	1860	1655
23	Hypothetical protein	2247	1588	1823	2693	1858	1656
24	G-D-S-L family lipolytic protein	-	1589	1822	2692	1857	1657
25	AraC family transcriptional regulator	-	-	-	-	1856	1658
26	Integral membrane sensor signal transduction histidine kinase	-	-	-	-	1855	1659
27	Family 1 extracellular solute-binding protein	-	-	-	-	1854	1660
28	Binding-protein-dependent transporters inner membrane component	-	-	-	-	1853	1661
29	ABC transporter permease	-	-	-	-	1852	1662
30	Arabinofuranosidase/xylosidase	-	1564	-	-	1851	-
31	Xylose isomerase	2243	1565	1818	2688	1850	1664
32	Xylulokinase	2242	1566	1817	2687	1849	1665

### Cellobiose and fructose metabolism

Cellobiose and fructose are utilized by all six strains via dedicated phosphotransferase system (PTS) transporter systems. In all six strains, orthologous clusters code for three-component phosphotransferase system (PTS) transporter systems that uses phosphoenolpyruvate to transport the sugar into the cell and phosphorylate it, generating intracellular fructose-1-phosphate or cellobiose-6-phosphate. A MerR family transcriptional regulator controls cellobiose uptake in all six strains. The six cellobiose utilization clusters also contain a gene coding for 6-phospho-β-glucosidase, which converts cellobiose-6-phosphate to glucose and glucose-6-phosphate. A DeoR family transcriptional regulator controls fructose uptake in all six strains. The six fructose utilization clusters also contain a gene coding for 1-phosphofructokinase, which converts fructose-1-phosphate to fructose-1,6-diphosphate.

### Carbohydrate clusters found outside the ~200 Kb region

#### Starch metabolism

Two separate gene clusters are dedicated to degradation of starch, one targeting α-1,4-linked glucooligosaccharides, and one targeting α-1,6-linked glucooligosaccharides. Genomic analysis indicates that five of the six strains (YS93 being the exception) possess the ability to degrade α-1,4-linked starch and starch-derived α-1,4-linked glucans. In the five strains, an orthologous cluster codes for a secreted α-amylase, a three-component ABC transporter system, and an intracellular α-amylase, all under the control of a LacI family transcriptional regulator (Table 9). The secreted α-amylase, transcriptional regulator and the three-component ABC transporter system show >90% identity among the five strains. The intracellular α-amylase genes of strains MC52, 12MC61, C56T3 and 56T2 code for 588 a.a. proteins with >90% identity to each other, but in 1MC16, the gene is truncated, coding for a 297 a.a. protein corresponding to the N-terminal domain of the 588 a.a. protein. In addition to the six-gene cluster, strains MC52, MC61, C56T3, and 56T2 possess an identical, two-gene insert containing a different secreted α-amylase (amyS) and a secreted amylopullulanase, located far downstream from the starch cluster. The utilization of three distinct secreted enzymes for degradation of starch is a highly unusual strategy for these *Geobacillus* species. In contrast, these *Geobacillus* species degrade xylan and arabinan using one secreted enzyme each, and no other secreted polysaccharide-degrading metabolic enzymes are secreted. None of the six strains contain the *Geobacillus* high molecular weight amylase that associates with the S-layer (Ferner-Ortner-Bleckmann et al., 2009), or the *Geobacillus* maltose-producing high molecular weight amylase (Diderichsen and Christiansen, 1988).

**Table 9:** **α-1,4-linked Glucooligosaccharide metabolic cluster**.

**Annotation**	**YS93**	**1MC16**	**MC52**	**MC61**	**56T2**	**56T3**
α-amylase (cyclomaltodextrinase)	-	0573	0632	1510	0721	2858
Extracellular solute-binding protein family 1	-	0572	0633	1511	0722	2857
Binding-protein-dependent transport systems inner membrane component	-	0571	0634	1512	0723	2856
Binding-protein-dependent transport systems inner membrane component	-	0570	0635	1513	0724	2855
Secreted α-amylase	-	0569	0636	1514	0725	2854
Transcriptional regulator, LacI family	-	0568	0637	1515	0726	2853
Secreted amylopullulanase	-	-	3302	3272	2870	3189
Secreted α-amylase (AmyS)	-	-	3303	3273	2871	3190

In all six strains, an orthologous cluster codes for a three-component ABC transporter system, and an intracellular α-1,6-glucosidase, all under the control of a LacI family transcriptional regulator. The transcriptional regulator and the three-component ABC transporter system show >90% identity among the six strains, while the α-1,6-glucosidase shows a lower identity (70%). The cluster may act synergistically with the starch cluster to take up and degrade the branched regions of partially degraded amylopectin, or the cluster may take up and degrade more highly branched substrates such as pullulan or glycogen fragments.

#### Galactose and galactoside utilization

The six strains each show distinct metabolic capabilities for galactose utilization (Table 10). All six strains utilize galactose via the Leloir pathway of GalK, GalT, and GalE (Holden et al., 2003), similar to the pathway in most organisms including *B. subtilis* (Chai et al., 2012). The pathway in all six strains is under the control of a LacI family transcriptional regulator. C56T3 possesses only the Leloir pathway and no transporter or galactosidase genes, suggesting a limited ability to utilize exogenous galactose or galactans. 1MC16 lacks transporter genes, but possesses a single β-galactosidase, suggesting 1MC16 is able to utilize galactose linked to xylan or arabinan that was transported into the cell via xylan or arabinan transporter systems. Similarly, strain YS93 lacks transporter genes, but possesses a single intracellular α-galactosidase, suggesting 1MC16 is able to utilize galactose linked to sucrose, xylan or arabinan that was transported into the cell via the corresponding transporter system. 56T2 possesses transporter genes and genes for two intracellular β-galactosidases, suggesting the ability to utilize lactose and galactan oligosaccharides. Finally, strains MC52 and MC61 possess transporter genes and genes for two intracellular β-galactosidases and one intracellular α-galactosidase, suggesting the ability to utilize a wide range of galactose-containing oligosaccharides. None of the strains possess the extracellular α-galactosidase identified in one strain of *G. stearothermophilus* (Talbot and Sygusch, 1990). The intracellular α-galactosidases show significant differences in sequence. The intracellular α-galactosidases of MC52 and MC61 share 100% identity with each other and 98% identity with the *G. stearothermophilus* α-galactosidase identified as AgaA (Merceron et al., 2012). The intracellular α-galactosidase of YS93 shares only 81–82% identity with *G. stearothermophilus* AgaA and theα-galactosidases of MC52 and MC61, but shares 93% identity with the *G. stearothermophilus* α-galactosidase identified as AgaN (Fridjonsson et al., 1999).

**Table 10:** **Galactose and galactoside metabolic cluster**.

**Annotation/corresponding gene**	**YS93**	**1MC16**	**MC52**	**MC61**	**56T2**	**56T3**
α-galactosidase	1518	-	2132	0528	-	-
Uncharacterized protein	1519	-	2131	0529	-	-
β-galactosidase, GH42	-	-	2130	0530	2119	-
Extracellular solute-binding protein family 1	-	-	2129	0531	2118	-
Binding-protein-dependent transport systems inner membrane component	-	-	2128	0532	2117	-
Binding-protein-dependent transport systems inner membrane component	-	-	2127	0533	2116	-
β-galactosidase, GH2	-	1068	2126	0534	2115	-
Galactokinase, GalK	1520	1066	2124	0536	2113	1361
UDP-glucose 4-epimerase, GalE	1521	1065	2123	0537	2112	1362
Gal-1-phosphate uridylyltransferase, GalT	1522	1064	2122	0538	2111	1363
Transcriptional regulator, LacI family	1523	1063	2121	0539	2110	1364

The MC52 and MC61 β-galactosidase, GH42 share 99% identity with the *G. stearothermophilus* β-galactosidase GanB (Solomon et al., 2013), while the 56T2 shares 96% identity with the *G. stearothermophilus* enzyme. The second β-galactosidase, β-galactosidase GH2 of MC52 and MC61 share 100% identity with each other and 96% identity with the 56T2 enzyme. The gene for this β-galactosidase appears to be uncommon among thermophiles, being identified only in the genome of *Geobacillus* sp. Strain WSUCF1 (Bhalla et al., 2013) (99% identity to MC52 and MC61) and *Anoxybacillus flavithermus* TNO-09.006 (Caspers et al., 2013) (98% identity to MC52 and MC61). This GH2 β-galactosidase is related to similar enzymes in mesophilic species such as *B. halodurans* strain ATCC BAA-125 (Takami et al., 2000) (69% identity to MC52 and MC61) and *Paenibacillus polymyxa* strain CR1 (Eastman et al., 2014) (67% identity to MC52 and MC61).

#### Sucrose metabolism

Sucrose is utilized by three of the six strains (MC52, MC61, and YS93) via a dedicated phosphotransferase system (PTS) transporter system. In all three strains, orthologous clusters code for three-component phosphotransferase system (PTS) transporter systems that uses phosphoenolpyruvate to transport the sugar into the cell and phosphorylate it, generating intracellular sucrose-6-phosphate under control of a MtlR family transcriptional regulator. The three sucrose utilization clusters also contain a gene coding for sucrose-6-phosphate hydrolase, which converts sucrose-6-phosphate to fructose and glucose-6-phosphate. The remaining three strains have no sucrose uptake system of any kind.

## Discussion

In this work we report the whole genome sequences of six new xylanolytic *Geobacillus* strains along with the genomic analysis of their capability to degrade carbohydrates. The six sequenced *Geobacillus* strains described here have a range of GC contents from 43.9 to 52.5%. Based on phylogenetic analysis, three of the strains, MC52, MC61, and 56T3 may be members of a single new species, and 56T2 may also be a member of a new species. The remaining two strains clade with named *Geobacillus* species (Zeigler, 2005).

Whole genome sequencing and analysis of these six strains gives a first look at the wide range of carbohydrate degradation capabilities (Table 11) of *Geobacillus* species. All six strains are predicted to utilize fructose, arabinose, xylose, mannitol, gluconate, xylan, and pullulan (α-1,6-glucosides). The gene clusters have identical organization and the individual proteins have a high percent identity to their homologs. Significant differences exist in the ability of the sequenced strains to utilize inositol, sucrose, lactose, α-mannosides, α-1,4-glucosides and arabinan. None of the strains was able to utilize all of these carbohydrates. Complete or partial utilization pathways were present or were completely absent in a strain-specific pattern. The proteins utilized in degradation of these carbohydrates showed greater strain-to-strain variation than the proteins utilized in degradation of fructose, arabinose, xylose, mannitol, gluconate, xylan, and pullulan.

**Table 11:** **Summary of carbohydrate utilization capabilities**.

	**YS93**	**1MC16**	**MC52**	**MC61**	**56T2**	**56T3**
Fructose	PTS	PTS	PTS	PTS	PTS	PTS
Arabinose	ABC	ABC	ABC	ABC	ABC	ABC
Xylose	ABC	ABC	ABC	ABC	ABC	ABC
Galactose	ABC	ABC	ABC	ABC	ABC	-
Gluconate	PER	PER	PER	PER	PER	PER
Inositol	-	PTS	-	-	-	-
Mannitol	PTS	PTS	PTS	PTS	PTS	PTS
Cellobiose	PTS	PTS	PTS	PTS	PTS	PTS
Sucrose	PTS	-	PTS	PTS	-	-
Lactose	-	-	ABC	ABC	-	-
Starch	-	ABC	ABC	ABC	ABC	ABC
α-Mannosides	-	ABC	-	-	-	-
Arabinan	-	-	ABC	ABC	-	-
Xylan	ABC	ABC	ABC	ABC	ABC	ABC
Panose/pullulan	ABC	ABC	ABC	ABC	ABC	ABC

*Gene clusters for utilization of listed substrates as described in text. ABC, three component ABC transporter system; PTS, three component phosphotransferase system; PER, permease system*.

Our group has sequenced and analyzed the genomes of a number of biomass degraders including three *Cellulomonas* spp. (Christopherson et al., 2013), *Bacillus cellulosilyticus* (Mead et al., 2013), *Fibrobacter succinogenes* (Brumm et al., 2011b; Suen et al., 2011) and *Dictyoglomus turgidum* (Brumm et al., 2011a). Comparison of the genomes of these biomass degraders to the six Geobacillus spp., show three major differences between the strategies employed by the *Geobacillus* and these other diverse organisms.

The *Geobacillus* spp. in this work were selected for their ability to hydrolyze MUX or MUC. Based on enzymatic assays, all six strains were able to utilize xylan, but only two strains, MC52 and MC61, were able to utilize arabinan. The genes for these activities were found in a large, conserved pentosan degradation cluster. Five of the six pentosan clusters include a region involved in arabinan degradation and all six include a region for xylan degradation, with over 50 possible genes in the combined pentosan cluster. The organization of the genes within the cluster is highly conserved in all the *Geobacillus* strains studied, and more importantly, none of the genes involved in pentosan metabolism are found outside this cluster. In the six diverse biomass degraders, pentosan degradation genes are not clustered, but are distributed randomly throughout the genomes. Random distributions of pentosan degradation genes are seen in other biomass degraders such as *Bacillus*, *Clostridium*, and *Streptomyces* species. These observations suggest that the large, single pentosan degradation cluster appears to be a unique feature of *Geobacillus* spp. The evolutionary advantages of a single cluster versus a random distribution are unclear, but suggest a single cluster may be an adaptation to life under extreme conditions. The *Geobacillus* pentosan degradation cluster is part of a ~200 kb unique super-cluster, containing five to eight distinct carbohydrate degradation clusters in a single genomic region, a feature not seen in other sequenced strains in related genera.

The *Geobacillus* spp. are also unique in their dependence on a minimum number of secreted enzymes for utilization of carbohydrates. Only two secreted enzymes, a xylanase and an arabinanase, are used in degradation of xylan and arabinan. Starch degradation utilizes three secreted enzymes. None of the *Geobacillus* spp. secrete xylosidases or arabinofuranosidases. In contrast to the *Geobacillus* spp., most other Gram-positive pentosan-degraders secrete multiple xylanases as well as multiple xylosidases. For example, *Cellulomonas flavigena* secretes 19 xylanases and 3 xylosidases, *Cellulomonas fimi* secretes 6 xylanases and 4 xylosidases, and *Cellulomonas gilvus* secretes 6 xylanases and 5 xylosidases (Christopherson et al., 2013). In further contrast to the *Geobacillus* spp., many other Gram-positive pentosan-degraders secrete combinations of other biomass-degrading enzymes such as cellulases, mannanases, xyloglucanases, pectinases, and pectate lyases. The genomes of the *Geobacillus* spp. lack orthologs of these secreted enzymes, indicating that *Geobacillus* spp. may target a limited range of carbohydrate polymers in intact biomass, or degrade biomass as part of a thermophilic consortium whose other members possess these activities.

Another unique feature of the *Geobacillus* pentosan cluster enzymes is the lack of targeting by attached carbohydrate binding modules (CBM) (Lombard et al., 2014). CBM modules are believed to improve enzyme efficiency by providing specific non-catalytic binding to the correct substrate (Boraston et al., 2004). CBM modules are present in many of the xylanases produced by thermophilic Gram-positive organisms including *Clostridium thermocellum* and *Caldicellulosiruptor* species (http://www.cazy.org/) (Lombard et al., 2014). The lack of CBM modules may indicate that the *Geobacillus* enzymes predate the evolution of CBM modules. Alternately, the lack of CBM modules make give *Geobacillus* enzymes the ability to utilize a broader range of substrates at the cost of a slower rate of hydrolysis.

The sequencing and genomic analysis of these six *Geobacillus* spp. confirms the belief that *Geobacillus* spp. are an excellent source of a variety of thermophilic enzymes with industrial applications. The variety of enzymes observed in a number of pathways, as well as the absence of previously identified Geobacillus enzymes such as the maltogenic (Diderichsen and Christiansen, 1988) and high molecular weight (Ferner-Ortner-Bleckmann et al., 2009) amylases suggest that sufficient genetic variability exists with the genus to supply additional new enzymes with novel applications.

### Conflict of interest statement

The authors are employees and shareholders of C5-6 Technologies (WI, USA), a company that creates bio-based solutions to efficiently convert biomass into five and six carbon sugars. The authors have no other relevant affiliations or financial involvement with any organization or entity with a financial interest in or financial conflict with the subject matter or materials discussed in the manuscript apart from those disclosed. No writing assistance was utilized in the production of this manuscript.
